# Free Vibration Analysis of Closed Moderately Thick Cross-Ply Composite Laminated Cylindrical Shell with Arbitrary Boundary Conditions

**DOI:** 10.3390/ma13040884

**Published:** 2020-02-17

**Authors:** Dongyan Shi, Dongze He, Qingshan Wang, Chunlong Ma, Haisheng Shu

**Affiliations:** 1 College of Mechanical and Electrical Engineering, Harbin Engineering University, Harbin 150001, China; shidongyan@hrbeu.edu.cn (D.S.); hdz2012071506@126.com (D.H.); shuhaisheng@hrbeu.edu.cn (H.S.); 2 State Key Laboratory of High Performance Complex Manufacturing, Central South University, Changsha 410083, China; 3 Department of Automotive Engineering, Harbin Vocational & Technical College, Harbin 150001, China

**Keywords:** wave based method, moderately thick composite laminated cylindrical shell, free vibration, arbitrary conditions

## Abstract

A semi-analytic method is adopted to analyze the free vibration characteristics of the moderately thick composite laminated cylindrical shell with arbitrary classical and elastic boundary conditions. By Hamilton’s principle and first-order shear deformation theory, the governing equation of the composite shell can be established. The displacement variables are transformed into the wave function forms to ensure the correctness of the governing equation. Based on the kinetic relationship between the displacement variables and force resultants, the final equation associated with arbitrary boundary conditions is established. The dichotomy method is conducted to calculate the natural frequencies of the composite shell. For verifying the correctness of the present method, the results by the present method are compared with those in the pieces of literatures with various boundary conditions. Furthermore, some numerical examples are calculated to investigate the effect of several parameters on the composite shell, such as length to radius ratios, thickness to radius ratios and elastic restrained constants.

## 1. Introduction

With the rapid development of the industry, composite laminated materials are increasingly used. The composite laminated cylindrical shell is one of the principal structural components and is widely used in various engineering applications, such as naval equipment, vehicle engineering, aerospace, and basic industries. In the past few decades, the dynamic analysis of composite shells has made considerable progress. People are paying more and more attention to developing more accurate and effective mathematical models and analyzing their dynamic behavior. Some researchers have proposed some of the classical and improved theories, also, different calculation methods are developed. The extensive researches are evolved by Lessia [1], Qatu [2,3,4,5], Reddy [6], Carrera [7,8], Ye [9] and others [10,11,12].

According to the previously reported studies, there are three main shell theories that are usually known: classical shell theory (CST) [13,14,15,16], first-order shear deformation shell theory (FSDST) [17,18,19,20,21] and higher-order shear deformation shell theory (HSDST) [22,23,24,25,26]. The classical shell theory is the basic theory, the transverse normal and shear deformations are ignored. Also, some theories are developed based on CST, such as Flügge’s theory and Donner–Mushtari’s theory. When anticipating the effects of transverse shear deformations, the FSDST is conducted. The transverse shear stiffness is corrected by the shear correction factor. HSDST analyzes the shell dynamic problem more precisely, but the amount of calculation is large. With continuous development in recent years, many researchers have conducted in-depth research on the dynamic analysis of the moderately thick composite laminated cylindrical shells. In this paper, some research statuses are listed. Alijani and Aghdam [27] proposed the Kantorovich method to investigate the moderately thick laminated cylindrical panels with several boundary conditions (i.e., F-F, C-C, and S-S). The loadings are set as uniform and sinusoidally distributed forms. Hosseini-Hashemi et al. [28] presented the state space method to study the free vibration characteristics of the rotating functionally graded circular cylindrical shell. The Sanders shear deformation theory, Coriolis, centrifugal and initial hoop tension effects are adopted to establish the motion equations. Sakka et al. [29] proposed the double Fourier series expansion method to analyze the free vibration characteristics of the moderately thick orthotropic cylindrical shell panels. The boundary condition is set as clamped and the Sanders kinematics is assembled to get the governing differential equations. Hao et al. [30] extended the isogeometric method [31] to study the buckling characteristics of the complex composite shells. Zhu et al. [32] conducted the modified Fourier series method to discuss the free vibration of the functionally graded open shells. The moderately thick shell forms are given as cylindrical, conical and spherical shells. Kurtaran [33] extended the Generalized Differential Quadrature (GDQ) method to study the transient characteristics of the moderately composite shallow shell. Maleki et al. [34] presented the GDQ method to investigate the static characteristics of moderately thick laminated cylindrical shell panels with different loadings and boundary conditions. The GDQ technique and Newmark’s plan are adopted to establish the governing equations. Fazilati and Ovesy [35] extended the spline method to discuss the parametric stability and instability region problem. The Koiter–Sanders theory is considered to express the linear strain terms when the shell structure is under harmonic in-plane loads. Tabiei and Simitses [36] analyzed the classical, first-order and higher-order shear deformation, the Donnell and Sanders type kinematics relations to describe the kinematic relations and equilibrium equations. Garcia et al. [37] investigated the effect of polycaprolactone nanofibers on the dynamic behavior of glass fiber reinforced polymer composites. Garcia et al. [38] conducted the influence of the inclusion of nylon nanofibers on the global dynamic behavior of glass fiber reinforced polymer (GFRP) composite laminates.

The wave based method (WBM) is a new analysis method to investigate the dynamic characteristics of the engineering structures. In recent years, some applications for WBM methods have gradually been developed. Yang et al. [39] analyzed the power flow of the plate structure by WBM. The results were compared with Finite element method (FEM) to validate the advantage of the present method. Koo et al. [40] proposed the WBM to discuss the semi-coupled structural–acoustic problem. He et al. [41] discussed the modeling acoustic problems and applied to the low-frequency applications. Also, the vibration characteristics of some engineering structures were extended by the WBM in engineering geometry applications, such as cylindrical shells with discontinuity in thickness [42], ring-stiffened cylindrical shells [43], composite laminated cylindrical shells [44], composite laminated shallow shells [45], cylindrical shells with non-uniform stiffener distribution [46], underwater cylindrical shells with bulkheads [47] and some coupled structures [48]. However, it can be seen that there is currently no relevant literature on the study of free vibration characteristics for moderately thick composite laminated cylindrical shells with arbitrary boundary conditions. Therefore, it is worthwhile to take advantage of the present method.

This paper aims to develop a new semi-analyzed method to investigate the free vibration characteristics of moderately thick composite laminated cylindrical shell with arbitrary boundary conditions. FSDST is adopted to describe the relationship between the displacement variables and transverse rotations. According to the Hamilton principle, the governing equation of the moderately thick composite laminated cylindrical shell is obtained. Transform the displacement variables into wave function forms to verify the motion governing equations. The total matrix is established according to boundary matrices that depend on arbitrary boundary conditions. To test and verify the free vibration characteristics of the moderately thick composite laminated cylindrical shell under arbitrary boundary conditions, the results by the present method are contrasted with the solutions in recent pieces of literature. Furthermore, some numerical examples are shown to discuss the effect of geometric parameters, stiffness constants and some conclusions are obtained. The advantage of this method is that it is easy to construct a global matrix, which is adapted to various boundary conditions, and has high calculation efficiency and high accuracy.

## 2. Theoretical Formulations

### 2.1. The Description of the Model

**Figure 1:**
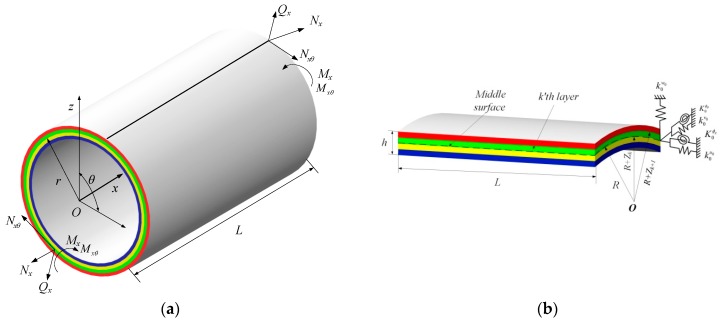
The schematic diagram of the moderately thick composite laminated cylindrical shell with elastic boundary conditions: (**a**) the whole composite shell; (**b**) the cross-section view of the composite shell.

Consider the model in Figure 1, the moderately thick composite laminated cylindrical shell with general boundary conditions. *L*, *R*, and *h* denote the length, mean radius and thickness of the shell. The global coordinate (*x*, *θ*, *z*) are set, the *x*, *θ* and *z* axes are taken in the axial circumferential and radial directions. In the *k*’th layer, the included angle of the composite material and principle direction is defined as *β*. The distances from the top and bottom surfaces to the middle surface are defined as *Z_k_*_+1_ and *Z_k_*. The middle surface displacements of the composite shell are defined as *u*_0_, *v*_0_, and *w*_0_, their directions are set in the *x*, *θ* and *z* axes. The transverse rotations about the *θ* and *x* axes are represented as *ϕ_x_* and *ϕ_θ_*. There are five groups of linear distribution and rotational springs and each ends.

### 2.2. Kinematic Relations and Stress Resultants

Through the description of the moderately thick composite laminated cylindrical shell, the displacement resultant of the shell is shown by the middle surface displacements and rotation variables, expressed as [2,49,50,51,52,53,54,55]:

(1) $$\begin{array}{l} u\left(x,\theta ,z,t\right)=u_{0}\left(x,\theta ,t\right)+z\phi_{x}\left(x,\theta ,t\right)\\ v\left(x,\theta ,z,t\right)=v_{0}\left(x,\theta ,t\right)+z\phi_{\theta }\left(x,\theta ,t\right)\\ w\left(x,\theta ,z,t\right)=w_{0}\left(x,\theta ,z,t\right) \end{array}$$

where *u*_0_, *v*_0_, and *w*_0_ are the displacements of the middle surface in the axial, circumferential and radial directions, *ϕ_x_* and *ϕ_θ_* are the transverse normal rations of the *x* and *θ* axis. *t* represents the time variables. The relationship between the strains and curvature changes of the moderately thick composite laminates shell is defined as:

(2) $$\varepsilon_{xx}^{0}=\frac{\partial u_{0}}{\partial x},\varepsilon_{\theta \theta }^{0}=\frac{\partial v_{0}}{R\partial \theta }+\frac{w_{0}}{R},\gamma_{x\theta }^{0}=\frac{\partial v_{0}}{\partial x}+\frac{\partial u_{0}}{R\partial \theta },\chi_{xx}=\frac{\partial \phi_{x}}{\partial x},\chi_{\theta \theta }=\frac{\partial \phi_{\theta }}{R\partial \theta },\chi_{x\theta }=\frac{\partial \phi_{\theta }}{\partial x}+\frac{\partial \phi_{x}}{R\partial \theta }$$

where $\varepsilon_{xx}^{0}$, $\varepsilon_{\theta \theta }^{0}$, and $\varepsilon_{x\theta }^{0}$ are the strains in the middle surface. *χ_xx_*, *χ_θθ_* and *χ_xθ_* denote the curvature changes. So, the relationship between the strain and displacement of the *k*th layer is shown:

(3) $$\varepsilon_{xx}=\varepsilon_{xx}^{0}+z\chi_{xx},\varepsilon_{\theta \theta }=\varepsilon_{\theta \theta }^{0}+z\chi_{\theta \theta },\gamma_{x\theta }=\gamma_{x\theta }^{0}+z\chi_{x\theta },\gamma_{xz}=\frac{\partial w_{0}}{\partial x}+\phi_{x},\gamma_{\theta z}=\frac{\partial w_{0}}{R\partial \theta }-\frac{v_{0}}{R}+\phi_{\theta }$$

where *Z_k_* < *z* < *Z_k_*_+1_. Related to the Hooke’s law, the relationship between the strains and stresses is given as:

(4) $$\left\{\begin{array}{c} \sigma_{xx}\\ \sigma_{\theta \theta }\\ \tau_{\theta z}\\ \tau_{xz}\\ \tau_{x\theta } \end{array}\right\}=\left[\begin{array}{ccccc} \bar{Q_{11}^{k}} & \bar{Q_{12}^{k}} & 0 & 0 & \bar{Q_{16}^{k}}\\ \bar{Q_{12}^{k}} & \bar{Q_{22}^{k}} & 0 & 0 & \bar{Q_{26}^{k}}\\ 0 & 0 & \bar{Q_{44}^{k}} & \bar{Q_{45}^{k}} & 0\\ 0 & 0 & \bar{Q_{45}^{k}} & \bar{Q_{55}^{k}} & 0\\ \bar{Q_{16}^{k}} & \bar{Q_{26}^{k}} & 0 & 0 & \bar{Q_{66}^{k}} \end{array}\right]\left\{\begin{array}{c} \varepsilon_{xx}\\ \varepsilon_{\theta \theta }\\ \gamma_{\theta z}\\ \gamma_{xz}\\ \gamma_{x\theta } \end{array}\right\}$$

where $\bar{Q_{ij}^{k}}$ (*i*, *j* = 1, 2, 4, 5, 6) are the elastic properties of the material. Through the transform matrix, the transformation stiffness matrix of the composite shell is determined as:

(5) $$\left[\begin{array}{ccccc} \bar{Q_{11}^{k}} & \bar{Q_{12}^{k}} & 0 & 0 & \bar{Q_{16}^{k}}\\ \bar{Q_{12}^{k}} & \bar{Q_{22}^{k}} & 0 & 0 & \bar{Q_{26}^{k}}\\ 0 & 0 & \bar{Q_{44}^{k}} & \bar{Q_{45}^{k}} & 0\\ 0 & 0 & \bar{Q_{45}^{k}} & \bar{Q_{55}^{k}} & 0\\ \bar{Q_{16}^{k}} & \bar{Q_{26}^{k}} & 0 & 0 & \bar{Q_{66}^{k}} \end{array}\right]=T\;\left[\begin{array}{ccccc} Q_{11}^{k} & Q_{12}^{k} & 0 & 0 & 0\\ Q_{12}^{k} & Q_{22}^{k} & 0 & 0 & 0\\ 0 & 0 & Q_{44}^{k} & 0 & 0\\ 0 & 0 & 0 & Q_{55}^{k} & 0\\ 0 & 0 & 0 & 0 & Q_{66}^{k} \end{array}\right]\;T^{T}$$

where $Q_{ij}^{k}$ (*i*, *j* = 1, 2, 4, 5, 6) are the transformation stiffness constants associated with the stresses and strains. For the orthotropic material, the constants can be given as:

(6) $$Q_{11}^{k}=\frac{E_{1}}{1-\mu_{12}\mu_{21}},Q_{12}^{k}=\frac{\mu_{12}E_{2}}{1-\mu_{12}\mu_{21}}=Q_{21}^{k},Q_{22}^{k}=\frac{E_{2}}{1-\mu_{12}\mu_{21}},Q_{44}^{k}=G_{23},Q_{55}^{k}=G_{13},Q_{66}^{k}=G_{12}$$

where *E*_1_ and *E*_2_ are Young’s modulus of the *k*th layer in the principal directions. *μ*_12_ and *μ*_21_ are the Poisson’s rations. Furthermore, the relationship of the Poisson’s rations is governed by the equation *μ*_12_*E*_2_ = *μ*_21_*E*_1_. *G*_12_, *G*_13_ and *G*_23_ are the rigidity modulus. For the isotropic material, the material relationship of coefficients is *E* = *E*_1_ = *E*_2_, *G* = *G*_12_ = *E*_1_/(2 + 2*μ*_12_) and *G*_12_ = *G*_13_ = *G*_23_.

In Equation (5), **T** is the transformation matrix, which is obtained as:

(7) $$T=\left[\begin{array}{ccccc} m^{2} & n^{2} & 0 & 0 & -2mn\\ n^{2} & m^{2} & 0 & 0 & 2mn\\ 0 & 0 & m & n & 0\\ 0 & 0 & -n & m & 0\\ mn & -mn & 0 & 0 & m^{2}-n^{2} \end{array}\right]$$

where *m* and *n* are the direction coefficients in the *k*th layer. *m* and *n* are defined as *m* = cos(*β*), *n* = sin(*β*) and *β* is the included angle.

The integration of load-bearing stresses in the cross-section and in-plane applies a moment in the thickness direction, the force and moment resultants are shown as:

(8) $$\begin{array}{c} \left\{N_{x},N_{\theta },N_{x\theta },Q_{x},Q_{\theta }\right\}=\int_{z}\left\{\sigma_{xx},\sigma_{\theta \theta },\tau_{x\theta },\tau_{xz},\tau_{\theta z}\right\}dz=\sum_{k=1}^{N}\int_{Z_{k}}^{Z_{k+1}}\left\{\sigma_{xx},\sigma_{\theta \theta },\tau_{x\theta },\tau_{xz},\tau_{\theta z}\right\}dz\\ \left\{M_{x},M_{\theta },M_{x\theta }\right\}=\int_{z}\left\{\sigma_{xx},\sigma_{\theta \theta },\tau_{x\theta }\right\}zdz=\sum_{k=1}^{N}\int_{Z_{k}}^{Z_{k+1}}\left\{\sigma_{xx},\sigma_{\theta \theta },\tau_{x\theta }\right\}zdz \end{array}$$

where *N* is the amount of the layer. Submitting Equations (2)–(4) into Equation (8), the relationship between the force and moment resultants to the strains is obtained as [2,49]:

(9) $$\begin{array}{c} \left[\begin{array}{l} N_{x}\\ N_{\theta }\\ N_{x\theta }\\ M_{x}\\ M_{\theta }\\ M_{x\theta } \end{array}\right]=\left[\begin{array}{cc} \begin{array}{ccc} A_{11} & A_{12} & A_{16}\\ A_{21} & A_{22} & A_{26}\\ A_{16} & A_{26} & A_{66} \end{array} & \begin{array}{ccc} B_{11} & B_{12} & B_{16}\\ B_{21} & B_{22} & B_{26}\\ B_{16} & B_{26} & B_{66} \end{array}\\ \begin{array}{ccc} B_{11} & B_{12} & B_{16}\\ B_{21} & B_{22} & B_{26}\\ B_{16} & B_{26} & B_{66} \end{array} & \begin{array}{ccc} D_{11} & D_{12} & D_{16}\\ D_{21} & D_{22} & D_{26}\\ D_{16} & D_{26} & D_{66} \end{array} \end{array}\right]\;\left[\begin{array}{l} \varepsilon_{xx}^{0}\\ \varepsilon_{\theta \theta }^{0}\\ \gamma_{x\theta }^{0}\\ \chi_{xx}\\ \chi_{\theta \theta }\\ \chi_{x\theta } \end{array}\right]\\ \left[\begin{array}{c} Q_{\theta }\\ Q_{x} \end{array}\right]=K_{c}\left[\begin{array}{cc} A_{44} & A_{45}\\ A_{45} & A_{55} \end{array}\right]\;\left[\begin{array}{c} \gamma_{\theta z}\\ \gamma_{xz} \end{array}\right] \end{array}$$

where {*N_x_*, *N_θ_*, *N_xθ_*} are the normal and shear force resultants. {*M_x_*, *M_θ_*, *M_xθ_*} represent the bending and twisting moment resultants. {*Q_x_*, *Q_θ_*} denote the transverse shear force resultants. *K_c_* is the shear correction factor and is taken as 5/6 in this paper. According to [49], the shear correction factor is caused by the true transverse shear stress predicted based on the three-dimensional elastic theory. In Equation (9), *A_ij_*, *B_ij_* and *D_ij_* (*i*,*j* = 1,2,4,5,6) are the stretching stiffness coefficients, coupling stiffness coefficients and bending stiffness coefficients, which can be given as:

(10) $$A_{ij}=\sum_{k=1}^{N}\bar{Q_{ij}^{k}}\left(Z_{k+1}-Z_{k}\right),B_{ij}=\frac{1}{2}\sum_{k=1}^{N}\bar{Q_{ij}^{k}}\left(Z_{{}_{k+1}}^{2}-Z_{k}^{2}\right),D_{ij}=\frac{1}{3}\sum_{k=1}^{N}\bar{Q_{ij}^{k}}\left(Z_{{}_{k+1}}^{3}-Z_{k}^{3}\right).$$

For analysis of the certain cross-ply moderately thick composite laminated cylindrical shell, the coefficients *A*_16_ = *A*_26_ = *B*_16_ =*B*_26_ = *D*_16_ = *D*_26_ = 0.

### 2.3. Governing Equations

Based on the FSDST and Hamilton’s principle, the governing equations of moderately thick composite laminated shell can be obtained as [2,49]:

(11) $$\begin{array}{l} \frac{\partial N_{x}}{\partial x}+\frac{\partial N_{x\theta }}{R\partial \theta }=I_{0}\frac{\partial^{2}u_{0}}{\partial t^{2}}+I_{1}\frac{\partial^{2}\phi_{x}}{\partial t^{2}}\\ \frac{\partial N_{x\theta }}{\partial x}+\frac{\partial N_{\theta }}{R\partial \theta }+\frac{Q_{\theta }}{R}=I_{0}\frac{\partial^{2}v_{0}}{\partial t^{2}}+I_{1}\frac{\partial^{2}\phi_{\theta }}{\partial t^{2}}\\ \frac{\partial Q_{x}}{\partial x}+\frac{\partial Q_{\theta }}{R\partial \theta }-\frac{N_{\theta }}{R}=I_{0}\frac{\partial^{2}w_{0}}{\partial t^{2}}\\ \frac{\partial M_{x}}{\partial x}+\frac{\partial M_{x\theta }}{R\partial \theta }-Q_{x}=I_{1}\frac{\partial^{2}u_{0}}{\partial t^{2}}+I_{2}\frac{\partial^{2}\phi_{x}}{\partial t^{2}}\\ \frac{\partial M_{\theta }}{R\partial \theta }+\frac{\partial M_{x\theta }}{\partial x}-Q_{\theta }=I_{1}\frac{\partial^{2}v_{0}}{\partial t^{2}}+I_{2}\frac{\partial^{2}\phi_{\theta }}{\partial t^{2}} \end{array}$$

where

(12) $$\left\{I_{0},I_{1},I_{2}\right\}=\sum_{k=1}^{N}\int_{Z_{k}}^{Z_{k+1}}\rho_{k}\left\{1,z,z^{2}\right\}dz$$

in which *ρ_k_* is the density constant. By submitting Equations (2) and (9) into Equation (11), the governing equation of motion for the moderately thick cross-ply composite laminated cylindrical shell can be given as:

(13) $$\left[\begin{array}{lllll} L_{11} & L_{12} & L_{13} & L_{14} & L_{15}\\ L_{21} & L_{22} & L_{23} & L_{24} & L_{25}\\ L_{31} & L_{32} & L_{33} & L_{34} & L_{35}\\ L_{41} & L_{42} & L_{43} & L_{44} & L_{45}\\ L_{51} & L_{52} & L_{53} & L_{54} & L_{55} \end{array}\right]\left\{\begin{array}{l} u_{0}\\ v_{0}\\ w_{0}\\ \phi_{x}\\ \phi_{\theta } \end{array}\right\}=\left\{\begin{array}{l} 0\\ 0\\ 0\\ 0\\ 0 \end{array}\right\}$$

where *L_ij_* (*i*, *j* = 1, 2, 3, 4, 5) are the coefficients, which can be obtained as:

$$\begin{array}{l} L_{11}=A_{11}\frac{\partial^{2}}{\partial x^{2}}+\frac{A_{66}}{R^{2}}\frac{\partial^{2}}{\partial s^{2}}-I_{0}\frac{\partial^{2}}{\partial t^{2}},L_{12}=\frac{A_{12}}{R}\frac{\partial^{2}}{\partial x\partial s}+\frac{A_{66}}{R}\frac{\partial^{2}}{\partial x\partial s}\\ L_{13}=\frac{A_{12}}{R}\frac{\partial }{\partial x},L_{14}=B_{11}\frac{\partial^{2}}{\partial x^{2}}+\frac{B_{66}}{R^{2}}\frac{\partial^{2}}{\partial s^{2}}-I_{1}\frac{\partial^{2}}{\partial t^{2}},L_{15}=\frac{B_{12}}{R}\frac{\partial^{2}}{\partial x\partial s}+\frac{B_{66}}{R}\frac{\partial^{2}}{\partial x\partial s}\\ L_{21}=L_{12},L_{22}=A_{66}\frac{\partial^{2}}{\partial x^{2}}-\frac{A_{22}}{R^{2}}\frac{\partial^{2}}{\partial s^{2}}-\frac{K_{c}A_{44}}{R^{2}}+I_{0}\frac{\partial^{2}}{\partial t^{2}},L_{23}=\frac{\left(K_{c}A_{44}+A_{22}\right)}{R^{2}}\frac{\partial }{\partial s}\\ L_{24}=\frac{\left(B_{66}+B_{12}\right)}{R}\frac{\partial^{2}}{\partial x\partial s},L_{25}=\frac{K_{c}A_{44}}{R}+\frac{B_{22}}{R^{2}}\frac{\partial^{2}}{\partial s^{2}}+B_{66}\frac{\partial^{2}}{\partial x^{2}}-I_{1}\frac{\partial^{2}}{\partial t^{2}}\\ L_{31}=-L_{13},L_{32}=-L_{23},L_{33}=-\frac{A_{22}}{R^{2}}+\frac{A_{44}K_{c}}{R^{2}}\frac{\partial^{2}}{\partial s^{2}}+K_{c}A_{55}\frac{\partial^{2}}{\partial x^{2}}-I_{0}\frac{\partial^{2}}{\partial t^{2}}\\ L_{34}=\left(A_{55}K_{c}-\frac{B_{21}}{R}\right)\frac{\partial }{\partial x},L_{35}=\left(\frac{A_{44}K_{c}}{R}-\frac{B_{22}}{R^{2}}\right)\frac{\partial }{\partial s}\\ L_{41}=L_{14},L_{42}=L_{24},L_{43}=-L_{34},L_{44}=\frac{D_{66}}{R^{2}}\frac{\partial^{2}}{\partial s^{2}}+D_{11}\frac{\partial^{2}}{\partial x^{2}}-K_{c}A_{55}-I_{2}\frac{\partial^{2}}{\partial t^{2}},L_{45}=\frac{\left(D_{12}+D_{66}\right)}{R}\frac{\partial^{2}}{\partial x\partial s}\\ L_{51}=L_{15},L_{52}=L_{25},L_{53}=-L_{35},L_{54}=L_{45},L_{55}=\frac{D_{22}}{R^{2}}\frac{\partial^{2}}{\partial s^{2}}+D_{66}\frac{\partial^{2}}{\partial x^{2}}-K_{c}A_{44}-I_{2}\frac{\partial^{2}}{\partial t^{2}} \end{array}$$

### 2.4. Implementation of the WBM

For the general cross-ply moderately thick composite laminated cylindrical shell, the generalized displacements functions are set as in the wave function forms:

(14) $$\left\{\begin{array}{l} u_{0}\left(x,\theta ,t\right)\\ v_{0}\left(x,\theta ,t\right)\\ w_{0}\left(x,\theta ,t\right)\\ \phi_{x}\left(x,\theta ,t\right)\\ \phi_{\theta }\left(x,\theta ,t\right) \end{array}\right\}=\sum_{n=0}^{\infty }\left\{\begin{array}{l} U_{n}e^{ik_{n}x}\cos(n\theta )e^{-i\omega t}\\ V_{n}e^{ik_{n}x}\sin(n\theta )e^{-i\omega t}\\ W_{n}e^{ik_{n}x}\cos(n\theta )e^{-i\omega t}\\ \Phi_{xn}e^{ik_{n}x}\cos(n\theta )e^{-i\omega t}\\ \Phi_{\theta n}e^{ik_{n}x}sin(n\theta )e^{-i\omega t} \end{array}\right\}$$

where *k_n_* is the characteristics wave number in the axial directions. *U_n_*, *V_n_*, *W_n_*, *Φ_xn_*, *Φ_θn_* are the displacement amplitudes that are associated with the circumferential mode number *n*. *ω* is the circular frequency and *t* is the time variable. Submitting Equation (14) into Equation (13), the governing equations are:

(15) $$\left[\begin{array}{lllll} T_{11} & T_{12} & T_{13} & T_{14} & T_{15}\\ T_{21} & T_{22} & T_{23} & T_{24} & T_{25}\\ T_{31} & T_{32} & T_{33} & T_{34} & T_{35}\\ T_{41} & T_{42} & T_{43} & T_{44} & T_{45}\\ T_{51} & T_{52} & T_{53} & T_{54} & T_{55} \end{array}\right]\left\{\begin{array}{l} U_{n}\\ V_{n}\\ W_{n}\\ \Phi_{xn}\\ \Phi_{\theta n} \end{array}\right\}=\left\{\begin{array}{l} 0\\ 0\\ 0\\ 0\\ 0 \end{array}\right\}$$

where *T_ij_* (*i*, *j* = 1, 2, 3, 4, 5) is the coefficient elements of the matrix **T** which can be shown as:

(16) $$\begin{array}{l} T_{11}=-k_{n}{}^{2}A_{11}-\frac{n^{2}A_{66}}{R^{2}}+I_{0}\omega^{2},T_{12}=\frac{ink_{n}\left(A_{12}+A_{66}\right)}{R},T_{13}=\frac{ik_{n}A_{12}}{R}\\ T_{14}=-k_{n}{}^{2}B_{11}-\frac{n^{2}B_{66}}{R^{2}}+I_{1}\omega^{2},T_{15}=\frac{ink_{n}\left(B_{12}+B_{66}\right)}{R}\\ T_{21}=T_{12},T_{22}=A_{66}k_{n}{}^{2}+\frac{n^{2}A_{22}}{R^{2}}+\frac{A_{44}K_{c}}{R^{2}}-I_{0}\omega^{2},T_{23}=\frac{n\left(KcA_{44}+A_{22}\right)}{R^{2}}\\ T_{24}=\frac{ink_{n}\left(B_{12}+B_{66}\right)}{R},T_{25}=B_{66}k_{n}{}^{2}-\frac{K_{c}A_{44}}{R}+\frac{n^{2}B_{22}}{R^{2}}-I_{1}\omega^{2}\\ T_{31}=-T_{13},T_{32}=-T_{23},T_{33}=-A_{55}k_{n}{}^{2}K_{c}-\frac{n^{2}A_{44}K_{c}}{R^{2}}-\frac{A_{22}}{R^{2}}+I_{0}\omega^{2}\\ T_{34}=ik_{n}A_{55}K_{c}-\frac{ik_{n}B_{12}}{R},T_{35}=\frac{nK_{c}A_{44}}{R}-\frac{nB_{22}}{R^{2}}\\ T_{41}=-T_{14},T_{42}=-T_{24},T_{43}=T_{34}\\ T_{44}=D_{11}k_{n}{}^{2}+A_{55}K_{c}+\frac{n^{2}D_{66}}{R^{2}}-I_{2}\omega^{2},T_{45}=-\frac{ink_{n}\left(D_{12}+D_{66}\right)}{R}\\ T_{51}=T_{15},T_{52}=T_{25},T_{53}=-T_{35},T_{54}=-T_{45}\\ T_{55}=k_{n}{}^{2}D_{66}+K_{c}A_{44}+\frac{n^{2}D_{22}}{R^{2}}-I_{2}\omega^{2} \end{array}.$$

To ensure the equation has a non-trivial solution, it is necessary to eliminate the determinant of the coefficient matrix **T**. So, the governing equation of the axial wave number *k_n_* can be reduced as a tenth order polynomial equation, which can be shown as:

(17) $$b_{10}k_{n}^{10}+b_{8}k_{n}^{8}+b_{6}k_{n}^{6}+b_{4}k_{n}^{4}+b_{2}k_{n}^{2}+b_{0}=0.$$

Equation (17) is a fifth-order equation of $k_{n}^{2}$ and *b*_10_, *b*_8_, *b*_6_, *b*_4_, *b*_2_ and *b*_0_ are the coefficients which are determined by the coefficient matrix **T**. The detailed expression of the coefficients is too complex and it is not at the core of the theoretical part of this article. So, the authors ignored it to make the paper leaner. The roots of the equation are solved with ten characteristics roots, ±*k*_*n*,1_, ±*k*_*n*,2_, ±*k*_*n*,3_, ±*k*_*n*,4_, ±*k*_*n*,5_. Based on the characteristics roots, there is one set of basic solution resultants {*ξ_n,i_*, *η_n,i_*, 1, *χ_n,i_*, *ψ*_*n*,i_}*^T^* for the corresponding characteristics wave number ±*k_n,i_* (*i* = 1–5), which are defined as:

(18) $$\begin{array}{c} \xi_{n,i}={\left[\frac{\Delta_{1}}{\Delta }\right]}_{k_{n}=\pm k_{n,i}}\\ \eta_{n,i}={\left[\frac{\Delta_{2}}{\Delta }\right]}_{k_{n}=\pm k_{n,i}}\\ \chi_{n,i}={\left[\frac{\Delta_{4}}{\Delta }\right]}_{k_{n}=\pm k_{n,i}}\\ \psi_{n,i}={\left[\frac{\Delta_{5}}{\Delta }\right]}_{k_{n}=\pm k_{n,i}} \end{array}$$

where ∆, ∆*_i_* (*i* = 1, 2, 4, 5) are given as:

(19) $$\begin{array}{ll} \Delta ={\left|\begin{array}{llll} T_{11} & T_{12} & T_{14} & T_{15}\\ T_{21} & T_{22} & T_{24} & T_{25}\\ T_{41} & T_{42} & T_{44} & T_{45}\\ T_{51} & T_{52} & T_{54} & T_{55} \end{array}\right|}_{k_{n}=\pm k_{n,i}} & \Delta_{1}={\left|\begin{array}{llll} -T_{13} & T_{12} & T_{14} & T_{15}\\ -T_{23} & T_{22} & T_{24} & T_{25}\\ -T_{43} & T_{42} & T_{44} & T_{45}\\ -T_{53} & T_{52} & T_{54} & T_{55} \end{array}\right|}_{k_{n}=\pm k_{n,i}}\\ \Delta_{2}={\left|\begin{array}{llll} T_{11} & -T_{13} & T_{14} & T_{15}\\ T_{21} & -T_{23} & T_{24} & T_{25}\\ T_{41} & -T_{43} & T_{44} & T_{45}\\ T_{51} & -T_{53} & T_{54} & T_{55} \end{array}\right|}_{k_{n}=\pm k_{n,i}} & \Delta_{4}={\left|\begin{array}{llll} T_{11} & T_{12} & -T_{13} & T_{15}\\ T_{21} & T_{22} & -T_{23} & T_{25}\\ T_{41} & T_{42} & -T_{43} & T_{45}\\ T_{51} & T_{52} & -T_{53} & T_{55} \end{array}\right|}_{k_{n}=\pm k_{n,i}}\\ \Delta_{5}={\left|\begin{array}{llll} T_{11} & T_{12} & T_{14} & -T_{13}\\ T_{21} & T_{22} & T_{24} & -T_{23}\\ T_{41} & T_{42} & T_{44} & -T_{43}\\ T_{51} & T_{52} & T_{54} & -T_{53} \end{array}\right|}_{k_{n}=\pm k_{n,i}} & \end{array}.$$

So, the generalized displacement functions can be transformed as:

(20) $$\delta_{n}=Y_{n}\left(\theta \right)D_{n}P_{n}\left(x\right)W_{n}$$

where **δ***_n_* = {*u*_0_, *v*_0_, *w*_0_, *ϕ_x_*, *ϕ_θ_*}*^T^* means the generalized displacement resultant. **Y***_n_*(*θ*) = *diag*{cos(*nθ*), sin(*nθ*), cos(*nθ*), cos(*nθ*), sin(*nθ*)} is the modal matrix in the circumferential direction. **P***_n_*(*x*) = *diag* {*exp*(*jk*_*n*,1_), *exp*(*jk_n_*_,2_), …, *exp*(*jk_n_*_,*ns*_)} is the wave number matrix and *n_s_* is the number of the characteristics roots of Equation (17) and the value of it is 10. **W***_n_* = {*W_n_*_,1_, *W_n_*_,2_, …, *W_n_*_,*ns*_}*^T^* is the wave contribution factor resultant. **D***_n_* is the displacement coefficient matrix, which can be shown as:

(21) $$D_{n}=\left[\begin{array}{lllll} \xi_{n,1} & \xi_{n,2} & \cdots & \xi_{n,n_{s}-1} & \xi_{n,n_{s}}\\ \eta_{n,1} & \eta_{n,2} & \cdots & \eta_{n,n_{s}-1} & \eta_{n,n_{s}}\\ 1 & 1 & \cdots & 1 & 1\\ \chi_{n,1} & \chi_{n,2} & \cdots & \chi_{n,n_{s}-1} & \chi_{n,n_{s}}\\ \psi_{n,1} & \psi_{n,2} & \cdots & \psi_{n,n_{s}-1} & \psi_{n,n_{s}} \end{array}\right].$$

The generalized force and moment resultant **f***_n_* = {*N_x_*, *N_xθ_* + *M_xθ_*/*R*, *Q_x_*+∂*M_xθ_*/*R*∂*_θ_*, *M_x_*, *M_xθ_*}*^T^* can be obtained by Equations (9) and (20) as:

(22) $$f_{n}=Y_{n}\left(\theta \right)F_{n}P_{n}\left(x\right)W_{n}$$

where **F***_n_* is the force and moment coefficient matrix and the elements *F_n,ji_* (*j* = 1–5, *i* = 1–*ns*) are shown as:

(23) $$\begin{array}{l} F_{n,1i}=ik_{n,i}A_{11}\xi_{n,i}+\frac{nA_{12}}{R}\eta_{n,i}+\frac{A_{12}}{R}+ik_{n,i}B_{11}\chi_{n,i}+\frac{nB_{12}}{R}\psi_{n,i}\\ \begin{array}{l} F_{n,2i}=\left(-\frac{nA_{66}}{R}-\frac{nB_{66}}{R^{2}}\right)\xi_{n,i}+\left(ik_{n,i}A_{66}+\frac{ik_{n,i}B_{66}}{R}\right)\eta_{n,i}\\ +\left(\frac{nD_{66}}{R^{2}}-\frac{nB_{66}}{R}\right)\chi_{n,i}+\left(\frac{ik_{n,i}D_{66}}{R}+ikB_{66}\right)\psi_{n,i} \end{array}\\ \begin{array}{l} F_{n,3i}=-\frac{n^{2}B_{66}}{R^{2}}\xi_{n,i}+\frac{ink_{n,i}B_{66}}{R}\eta_{n,i}+ik_{n,i}K_{c}A_{55}\\ +\left(K_{c}A_{55}-\frac{n^{2}D_{66}}{R^{2}}\right)\chi_{n,i}+\frac{ink_{n,i}D_{66}}{R}\psi_{n,i} \end{array}\\ F_{n,4i}=ik_{n,i}B_{11}\xi_{n,i}+\frac{nB_{12}}{R}\eta_{n.i}+\frac{B_{12}}{R}+ik_{n,i}D_{11}\chi_{n,i}+\frac{nD_{12}}{R}\psi_{n,i}\\ F_{n,5i}=-\frac{nB_{66}}{R}\xi_{n,i}+ik_{n,i}B_{66}\eta_{n,i}-\frac{nD_{66}}{R}\chi_{n,i}+ik_{n,i}D_{66}\psi_{n,i} \end{array}.$$

For the classical boundary conditions, some boundary conditions are introduced as:

Free edge (*F*):
  (24) $$N_{x}=N_{x\theta }+\frac{M_{x\theta }}{R}\left(F_{1}\right)=M_{x}=M_{x\theta }=Q_{x}+\frac{\partial M_{x\theta }}{R\partial \theta }\left(F_{2}\right)=0.$$
Clamped edge (*C*):
  (25) $$u=v=w=\phi_{x}=\phi_{\theta }=0.$$
Simply-supported edge (*SS*):
  (26) $$u=v=w=M_{x}=\phi_{\theta }=0.$$
Shear-diaphragm edge (*SD*):
  (27) $$N_{x}=v=w=M_{x}=M_{x\theta }=0.$$

Also, the elastic boundary conditions can be given in some forms as: when the elastic restrained with the stiffness constant *K_u_* in the axial direction, the corresponding boundary equation can be shown as:

(28) $$\begin{array}{l} u:\begin{array}{c} x=0:K_{u}u_{0}\left(x,\theta ,t\right)-N_{x}\left(x,\theta ,t\right)=0\\ x=L:K_{u}u_{0}\left(x,\theta ,t\right)+N_{x}\left(x,\theta ,t\right)=0 \end{array}\\ v:\begin{array}{l} x=0:K_{v}v_{0}\left(x,\theta ,t\right)-F_{1}\left(x,\theta ,t\right)=0\\ x=L:K_{v}v_{0}\left(x,\theta ,t\right)+F_{1}\left(x,\theta ,t\right)=0 \end{array}\\ w:\begin{array}{c} x=0:K_{w}w_{0}\left(x,\theta ,t\right)-F_{2}\left(x,\theta ,t\right)=0\\ x=L:K_{w}w_{0}\left(x,\theta ,t\right)+F_{2}\left(x,\theta ,t\right)=0 \end{array}\\ \phi_{x}:\begin{array}{c} x=0:K_{\phi x}\phi_{x}\left(x,\theta ,t\right)+M_{x}\left(x,\theta ,t\right)=0\\ x=L:K_{\phi x}\phi_{x}\left(x,\theta ,t\right)-M_{x}\left(x,\theta ,t\right)=0 \end{array}\\ \phi_{\theta }:\begin{array}{c} x=0:K_{\phi \theta }\phi_{\theta }\left(x,\theta ,t\right)+M_{x\theta }\left(x,\theta ,t\right)=0\\ x=L:K_{\phi \theta }\phi_{\theta }\left(x,\theta ,t\right)-M_{x\theta }\left(x,\theta ,t\right)=0 \end{array} \end{array}$$

where *K_v_*, *K_w_*, *K_ϕx_*, *K_ϕθ_* are the corresponding stiffness constants in different displacements. For the combination of elastic boundary conditions, the boundary equations can refer to Equation (28). The total matrix **K** of the whole structure depends on the generalized displacement resultants, force resultants and boundary conditions. The expression of the total matrix **K** is:

(29) $$K=\left[\begin{array}{cc} B_{1}\left(0\right) & \\ D_{n}P_{n}\left(L\right) & -D_{n}P_{n}\left(0\right)\\ F_{n}P_{n}\left(L\right) & -F_{n}P_{n}\left(0\right)\\ & B_{2}\left(0\right) \end{array}\right]$$

where **D***_n_* and **F***_n_* are the displacement and force coefficient matrix; **P***_n_* is the wave number matrix and the positions are set as *x* = 0 and *x* = *L*. **B**_1_(*x*) and **B**_2_(*x*) are the boundary matrix which is related to the boundary conditions.

For the classical boundary conditions, the boundary matrix **B**_1_(*x*) and **B**_2_(*x*) are set as:

(30) $$B_{1,2}\left(x\right)=\left(T_{\delta }D_{n}+T_{f}F_{n}\right)P_{n}\left(x\right)$$

where **T***_δ_* and **T***_f_* are the transform matrices of the boundary matrix and the detailed expression of the transform vectors are:

Free edge (*F*):
  (31) $$\begin{array}{l} T_{\delta }=diag\left\{0,0,0,0,0\right\}\\ T_{f}=diag\left\{1,1,1,1,1\right\} \end{array}.$$
Clamped edge (*C*):
  (32) $$\begin{array}{l} T_{\delta }=diag\left\{1,1,1,1,1\right\}\\ T_{f}=diag\left\{0,0,0,0,0\right\} \end{array}.$$
Simply-supported edge (*SS*):
  (33) $$\begin{array}{l} T_{\delta }=diag\left\{1,1,1,0,1\right\}\\ T_{f}=diag\left\{0,0,0,1,0\right\} \end{array}.$$
Shear-diaphragm edge (*SD*):
  (34) $$\begin{array}{l} T_{\delta }=diag\left\{0,1,1,0,0\right\}\\ T_{f}=diag\left\{1,0,0,1,1\right\} \end{array}.$$

For the elastic boundary conditions, the boundary matrix **B**_1_(*x*) and **B**_2_(*x*) are given as:

(35) $$B_{1,2}\left(x\right)=\left(K_{\delta }D_{n}\pm F_{n}\right)P_{n}\left(x\right)$$

where **K***_δ_* is the stiffness transform matrix and the detailed expression is: when the elastic restrained with the stiffness constant *K_u_* in the axial direction, the stiffness transform matrix is given as:

(36) $$K_{\delta }=diag\left\{K_{u},0,0,0,0\right\}.$$

When the other directions are under elastic restrained, the stiffness matrices **K***_δ_* are given with different stiffness constants as:

(37) $$\begin{array}{ll} v: & K_{\delta }=diag\left\{0,K_{v},0,0,0\right\}\\ w: & K_{\delta }=diag\left\{0,0,K_{w},0,0\right\}\\ \phi_{x}: & K_{\delta }=diag\left\{0,0,0,K_{\phi x},0\right\}\\ \phi_{\theta }: & K_{\delta }=diag\left\{0,0,0,0,K_{\phi \theta }\right\} \end{array}.$$

When the composite shell is under the combination of elastic restrained, the boundary matrix **B**_1_(*x*) and **B**_2_(*x*) can refer to the Equations (36) and (37). To calculate the natural frequencies, the external force resultant **F** should vanish, and by searching the zero position of the total matrix **K** using the dichotomy method. In each of the circumferential mode numbers *n*, a series of determinant values of the total matrix **K** are calculated. The value of the experimental value is generated until the sign change occurs, and then the dichotomy method iteratively interpolates to locate the zero of the determinant.

## 3. Numerical Examples and Discussion

In this section, some examples are calculated to investigate the free vibration characteristics of the composite shell with classical, elastic, and their combination boundary conditions. Several numerical examples are accepted to verify the correctness of the present method.

### 3.1. Composite Laminated Cylindrical Shell with Classical Boundary Conditions

The composite shell under the classical boundary conditions is widely used in some engineering field applications and is also the focal point of many researchers. In this part, the dynamic analysis of this topic is analyzed.

**Figure 2:**
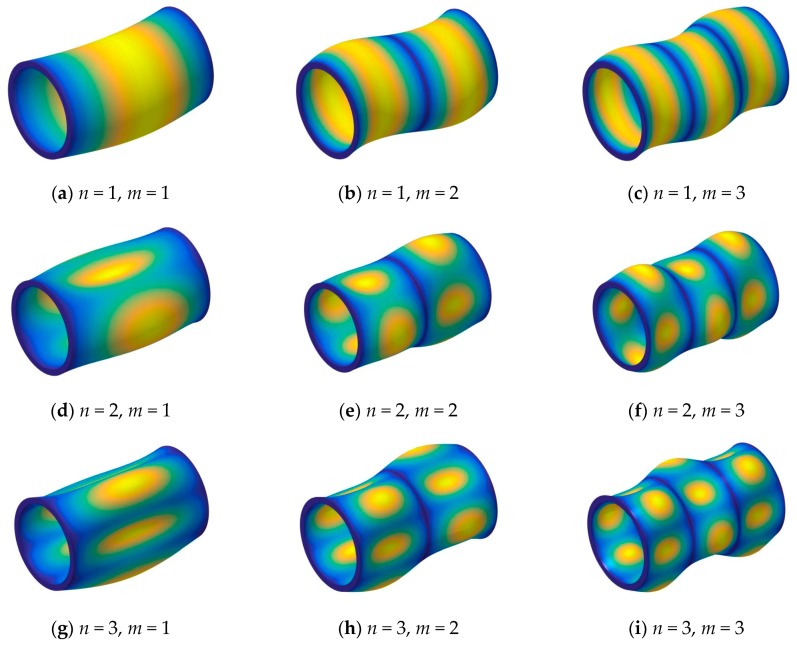

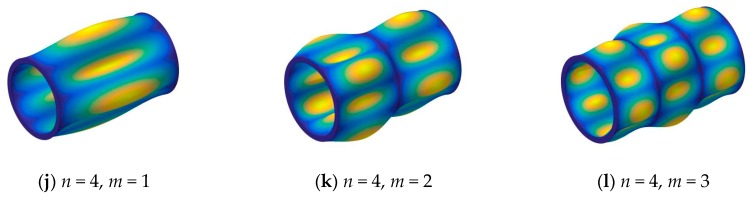
The modal shapes of a three-layered [0°/90°/0°] composite shell with simply-supported (S-S) boundary conditions.

First, in Table 1, the three layered [0°/90°/0°] composite shell under some classical boundary conditions is considered (i.e., F-F, S-S, C-C). The material properties and geometric parameters are given as: *R* = 1 m, *L*/*R* = 5, *h/R* = 0.05, *E*_2_ = 1 GPa, *E*_1_/*E*_2_ = 25, *μ*_12_ = 0.25, *G*_12_ = 0.5*E*_2_, *G*_13_ = 0.5*E*_2_, *G*_23_ = 0.2*E*_2_, *ρ* = 1700 kg/m^3^. The comparison of the frequency parameter $\Omega =\omega L^{2}\sqrt{\rho /E_{2}}/h$ is studied. The first four circumferential wave numbers (i.e., *n* = 1, 2, 3, 4) and the first longitudinal mode (i.e., *m* = 1) are calculated. The frequency parameters are compared with the results by Messia and Soldatos [56] and Jin et al. [57], from Table 1, the differences between the results by the present method and reported literatures are small, the maximum error is 3.01%. The differences are caused by different solution program methods. Furthermore, in each circumferential wave number, the maximum frequency parameters are under the boundary condition C-C, especially, when *n* = 1, the maximum frequency parameter is fixed under the boundary condition F-F. The reason is that the boundary conditions have a significant effect on the frequency parameters. In order to further investigate the free vibration characteristics of composite laminated cylindrical shells with arbitrary boundary conditions, some mode shapes (*n*, *m*) of the composite laminated cylindrical shell are shown in Figure 2.

The numerical examples in the previous studies considered the thin composite shell with various classical boundary conditions. To verify the correctness of the present method, more numerical examples are considered. In Table 2, the fundamental frequency parameter $\Omega =\omega L^{2}\sqrt{\rho /E_{2}}/100h$ of the moderately thick composite shell with the different length to radius ratios under four types of classical boundary conditions (i.e., S-S, S-C, C-C, C-F) are shown. There are two types of cross-ply laminated schemes (i.e., [0°/90°] and [0°/90°/0°]) and two kinds of length to radius ratios (i.e., *L*/*R* = 1, 2) are discussed. The results of the present method are compared with the results by Khdeir et al. [58], Thinh and Nguyen [59] and Jin et al. [57]. The geometric and material parameters are given as: *R* = 1 m, *h*/*R* = 0.2, *E*_2_ = 1 GPa, *E*_1_/*E*_2_ = 40, *μ*_12_ = 0.25, *G*_12_ = 0.6*E*_2_, *G*_13_ = 0.5*E*_2_, *G*_13_ = 0.5*E*_2_, *ρ* = 1600 kg/m^3^. From Table 2, the results of the present method agree well with the results in the literatures, the small differences are related to different shell theory and numerical methods. For solving the vibration characteristics of the moderately thick composite laminated cylindrical shell, the vibration characteristics of the whole system can be solved by the elastic equation: (**K**−ω^2^ × **M**) = 0, where **K** is the stiffness matrix for the shallow shell and **M** is the mass matrix, ω is the natural frequency for the moderately thick composite laminated cylindrical shell. Different boundary conditions cause the stiffness matrix to change. For the simply-supported (S-S) boundary condition, the determinant of the stiffness matrix becomes smaller compared to the clamped (C-C) boundary condition, and when the mass matrix remains unchanged, the natural frequency decreases. When the length to radius value changes from 1 to 2, the length quadratic variable in the frequency parameter $\Omega =\omega L^{2}\sqrt{\rho /E_{2}}/100h$ will be four times larger, and the frequency parameters are also increased. So, the effect of the length to radius ratios on the free vibration characteristics cannot be expressed.

Next, the effect of thickness to radius ratios on the frequency parameter is considered, the boundary condition is set as simply-supported. Two types of cross-ply laminated schemes (i.e., [0°/90°/90°/0°] and [0°/90°/90°/0°]) and three kinds of thickness to radius ratios (i.e., *h*/*R* = 0.1, 0.2, 0.3) are discussed. The material parameters and geometric constants are same as the previous example, the ratio of length to radius is given as *L*/*R* = 1. The frequency parameters of the three lowest natural frequencies $\Omega =\omega h\sqrt{\rho /G_{12}}/\pi$ are compared with the results in the literature that were investigated by Thinh [59] and Jin et al. [57]. From Table 3, the differences between the results of the present method and other results in the literature are small, and the differences are related to a variety of numerical methods and shell theories.

For analysis of the effect of length to radius ratios and thickness to radius ratios, one type of three-layered cross-ply [0°/90°/0°] composite laminated cylindrical shell with simply-supported and clamped boundary conditions is considered. The first longitudinal modal (i.e., *m* = 1) frequency parameter $\Omega =\omega R\sqrt{\rho /E_{2}}$ is calculated for different circumferential numbers (i.e., *n* = 1, 2, 3) with various thickness to radius ratios (i.e., *h*/*R* = 0.05–0.1), and length to radius ratios (i.e., *L*/*R* = 1–4) are calculated in Table 4 and Table 5. The material properties are given as: *E*_2_ = 2 GPa, *E*_1_/*E*_2_ = 25, *μ*_12_ = 0.25, *G*_12_ = 0.5*E*_2_, *G*_13_ = 0.5*E*_2_, *G*_23_ = 0.2*E*_2_, *ρ* = 1600 kg/m^3^. When studying the effect of the length to radius ratios, keeping material parameters and radius constant, the frequency parameters are only related to the natural frequency of the moderately thick composite laminated cylindrical shell. It can be seen from Table 4 and Table 5, with the growth of the length to the radius ratios *L*/*R*, the frequency parameter is generally decreased. Furthermore, the frequency parameter generally grows with the thickness to radius ratio increase. So, the effects of length to radius ratio and thickness to radius ratio are different from the frequency parameter of the moderately thick composite laminated cylindrical shell with simply-supported and clamped boundary conditions.

### 3.2. Composite Laminated Cylindrical Shell with Elastic Boundary Conditions

It is necessary and significant to study the vibration analysis of the composite laminated cylindrical shell under elastic restrained. Through the introducing of the elastic boundary conditions, the stiffness transform matrix is established by different elastic boundary conditions, in this paper, four types of typical elastic boundary conditions are considered:

Type 1 (EC1): axial displacement is under elastic restrained and the corresponding stiffness transform matrix **K***_δ_* is given as:

(38) $$K_{u}=10^{7},K_{\delta }=diag\left\{10^{7},0,0,0,0\right\}.$$

Type 2 (EC2): circumferential displacement is under elastic restrained and the corresponding stiffness transform matrix **K***_δ_* is given as:

(39) $$K_{v}=10^{7},K_{\delta }=diag\left\{0,10^{7},0,0,0\right\}.$$

Type 3 (EC3): radial displacement is under elastic restrained and the corresponding stiffness transform matrix **K***_δ_* is given as:

(40) $$K_{w}=10^{7},K_{\delta }=diag\left\{0,0,10^{7},0,0\right\}.$$

Type 4 (EC4): axial and circumferential displacements are under elastic restrained and the corresponding stiffness transform matrix **K***_δ_* is given as:

(41) $$K_{u}=K_{v}=10^{7},K_{\delta }=diag\left\{10^{7},10^{7},0,0,0\right\}.$$

**Figure 3:**
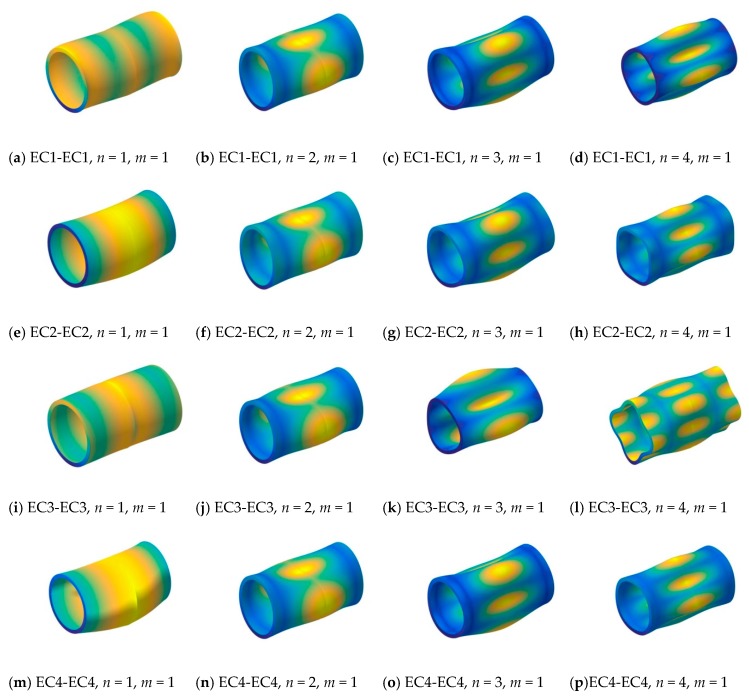
The modal shapes of a three-layered [0°/90°/0°] composite shell with various elastic boundary conditions.

First, two types—[0°/90°/0°] and [0°/90°]—of composite laminated cylindrical shells with classical and elastic boundary conditions (i.e., SD-SD, S-S, C-C, EC1-EC1, EC2-EC2, EC3-EC3, EC4-EC4) are discussed. The first longitudinal mode frequency parameter $\Omega =\omega L^{2}\sqrt{\rho /E_{2}}/h$ is calculated for various circumferential numbers (i.e., *n* = 1, 2, 3, 4). The material properties and geometric parameters are given as: *L*/*R* = 4, *h*/*R* = 0.1, *E*_2_ = 2 GPa, *E*_1_/*E*_2_ = 25, *μ*_12_ = 0.25, *G*_12_ = 0.5*E*_2_, *G*_13_ = 0.5*E*_2_, *G*_23_ = 0.2*E*_2_, *ρ* = 1500 kg/m^3^. The results calculated by the present method are compared with the solutions by Jin et al. [57] in Table 6 and Table 7. From the table, it is obvious that with different elastic boundary conditions for different layer-type composite shells, the highest frequency parameters are listed in the columns with elastic boundary condition EC1-EC1 in circumferential mode *n* = 1, and in the other circumferential mode *n* = 2, 3, 4, they appear in the columns with elastic boundary condition EC2-EC2. It is because the frequency parameter is related to the boundary condition and circumferential mode. In order to further investigate the free vibration characteristics of composite laminated cylindrical shells with elastic boundary conditions, some mode shapes (*n*, *m*) of the composite laminated cylindrical shell are shown in Figure 3.

Next, the effect of the stiffness constants is investigated. A three-layered cross-ply [90°/0°/90°] composite shell with complicated elastic boundary conditions is considered. The composite shell is under elastic restrained with one kind of spring stiffness in each displacement direction at one end; on the other end, the composite shell is under the simply-supported boundary condition. The first longitudinal mode (i.e., *m* = 1) frequency parameter $\Omega =\omega L^{2}\sqrt{\rho /E_{2}}/h$ is calculated for various circumferential numbers (i.e., *n* = 1, 2, 3, 4) with different elastic restrained *K_u_*, *K_v_*, *K_w_*, *K_ϕx_, K_ϕθ_*, which are calculated with various stiffness constants (i.e., 0–10^12^). The material parameters and geometric properties are given as: *L*/*R* = 4, *h*/*R* = 0.1, *E*_2_ = 2 GPa, *E*_1_/*E*_2_ = 25, *μ*_12_ = 0.25, *G*_12_ = 0.5*E*_2_, *G*_13_ = 0.5*E*_2_, *G*_23_ = 0.2*E*_2_, *ρ* = 1500 kg/m^3^. From Table 8, the frequency parameters are almost all in one certain value when the composite shell is only restrained by the rotation spring *K_ϕx_* and *K_ϕθ_*. When the composite shell is only restrained by the circumferential *K_v_* and radial spring *K_w_*, the frequency parameters generally increase with the changing of the stiffness constant. When the composite shell is only restrained by the axial spring *K_u_*, the frequency parameters have smaller growth with the increasing of the stiffness constants. It can be founded that the effect of circumferential spring *K_v_* and radial spring *K_w_* are more obvious than the other direction springs. When the circumferential wave number *n* = 1, the increase of the frequency parameters is larger than *n* = 2, 3. So, when the composite shell is under the S-elastic boundary condition, the effects of circumferential *K_v_* and radial spring *K_w_* are more obvious than the other direction springs.

Furthermore, the composite shell is considered under the S-elastic boundary condition in which only one displacement is under elastic restrained and other displacements are fixed. The frequency parameter, material constants and geometric properties are the same as the previous example. In Table 8, the frequency parameter $\Omega =\omega L^{2}\sqrt{\rho /E_{2}}/h$ is calculated. The expression of boundary matrix **B**_1_(*x*) and **B**_2_(*x*) are reduced as:

(42) $$B_{1,2}\left(x\right)=\left(K_{\delta }D_{n}\pm K_{f}F_{n}\right)P_{n}\left(x\right).$$

For different elastic boundary conditions, the corresponding stiffness transform matrices **K***_δ_* are given as:

(43) $$\begin{array}{ll} \mathrm{EC}1: & \{\begin{array}{l} K_{\delta }=diag\left\{K_{u},1,1,1,1\right\}\\ K_{f}=diag\left\{1,0,0,0,0\right\} \end{array}\\ \mathrm{EC}2: & \{\begin{array}{l} K_{\delta }=diag\left\{1,K_{v},1,1,1\right\}\\ K_{f}=diag\left\{0,1,0,0,0\right\} \end{array}\\ \mathrm{EC}3: & \{\begin{array}{l} K_{\delta }=diag\left\{1,1,K_{w},1,1\right\}\\ K_{f}=diag\left\{0,0,1,0,0\right\} \end{array}\\ \mathrm{EC}4: & \{\begin{array}{l} K_{\delta }=diag\left\{K_{u},K_{v},1,1,1\right\}\\ K_{f}=diag\left\{1,1,0,0,0\right\} \end{array} \end{array}.$$

In Table 9, the frequency parameters with different elastic restrained stiffness constants are calculated. It is obvious that with the changing of the stiffness constants from 0 to 10^12^, the frequency parameters are almost unchanged and remain in a certain range. So the effect of the elastic restrained stiffness constants for the S-elastic boundary condition, which is set as one displacement restrained and others are fixed of the composite shell, are small and the frequency parameters are almost all remaining in a stable range. So, for various elastic boundary condition combinations, the effects of the elastic spring restrained on the free vibration characteristics of moderately thick composite laminated cylindrical shells are different. In some cases, the effect of the elastic restrained springs is obvious. Also, the effect of the elastic restrained spring is not obvious in some numerical cases.

## 4. Conclusions

The wave base method is conducted to analyze the free vibration characteristics of moderately thick composite laminated cylindrical shells with arbitrary classical and elastic boundary conditions. According to the first-order shear deformation shell theory and Hamilton principle, the governing equation of the composite laminated shell is established. The displacement variables are transformed into wave function forms. Related to different boundary conditions, the boundary matrices are obtained to establish the total matrix. The natural frequencies are solved by the dichotomy method to experiment with the zero location of the total matrix determinant. For the wave based method, the advantage is that the boundary conditions are easy to replace. If the boundary conditions need to be changed, only the boundary condition matrix **B**_1_ and **B**_2_ need to be changed, including classical boundaries, elastic boundaries and their combined forms. To analyze the free vibration characteristics of moderately thick composite laminated shells, the solutions are easy to obtain in the wave function forms, and the shell structure does not need to be divided into shell segments. For the free vibration characteristics of the moderately thick composite laminated cylindrical shell with arbitrary boundary conditions, the solutions by the present method have better precision than the results in some reported literatures. Furthermore, some numerical examples are shown and the conclusions follow as:

First, the frequency parameters of moderately thick composite laminated cylindrical shells with arbitrary boundary conditions are calculated. Through the comparison of the results, it can be seen that the method proposed in this paper is more accurate for the calculation of the shell.

Second, the effect of the geometric constants, such as length to radius ratios and thickness to thickness ratios, on the frequency parameters are discussed. It is seen that different geometric constants have various effects on the frequency parameters.

Third, the influence of the boundary elastic restrained stiffness constants on the natural frequency parameters is discussed. The changing ranges of the elastic restrained stiffness constants in various directions are from 0–10^12^. From the variations of the natural frequency parameters, it can be concluded that the effect of the elastic restrained stiffness on the natural frequency parameters is not obvious. With the growth of the stiffness constants in various directions, the natural frequencies have a small range of fluctuations and are basically stable within a range.

## Figures and Tables

**Table 1:** Frequency parameters $\Omega =\omega L^{2}\sqrt{\rho /E_{2}}/h$ for a three-layer cross-ply cylindrical shell [0°/90°/0°] with various classical boundary conditions (*R* = 1 m, *L*/*R* = 5, *h/R* = 0.05, *E*_2_ = 1 GPa, *E*_1_/*E*_2_ = 25, *μ*_12_ = 0.25, *G*_12_ = 0.5*E*_2_, *G*_13_ = 0.5*E*_2_, *G*_23_ = 0.2*E*_2_, *ρ* = 1700 kg/m^3^; *m* = 1).

*n*	WBM	Ref. [56]	Error	Ref. [57]	Error
	F-F				
1	304.179	304.13	0.02%	304.16	0.01%
2	26.558	26.58	−0.08%	26.56	−0.01%
3	77.027	74.91	2.83%	74.78	3.01%
4	144.798	142.93	1.31%	142.51	1.61%
5	230.986	229.74	0.54%	228.7	1.00%
	**SD-SD**				
1	151.486	151.49	0.00%	151.49	0.00%
2	92.564	92.57	−0.01%	92.57	−0.01%
3	95.253	95.37	−0.12%	95.27	−0.02%
4	149.999	150.42	−0.28%	150.01	−0.01%
5	232.927	233.97	−0.45%	232.94	−0.01%
	**C-C**				
1	159.443	159.31	0.08%	159.44	0.00%
2	107.889	107.71	0.17%	107.89	0.00%
3	108.106	108.05	0.05%	108.11	0.00%
4	156.945	157.23	−0.18%	156.94	0.00%
5	236.764	237.7	−0.39%	236.76	0.00%

**Table 2:** Frequency parameters $\Omega =\omega L^{2}\sqrt{\rho /E_{2}}/100h$ for two types of cross-ply composite laminated cylindrical shell with different length to radius ratios and boundary conditions (*R* = 1 m, *h*/*R* = 0.2, *E*_2_ = 1 GPa, *E*_1_/*E*_2_ = 40, *μ*_12_ = 0.25, *G*_12_ = 0.6*E*_2,_ *G*_13_ = 0.5*E*_2_, *G*_13_ = 0.5*E*_2_, *ρ* = 1600 kg/m^3^).

Layer-Type	Shell Theories	S-S		S-C		C-C		C-F	
		*L*/*R* = 1	*L*/*R* = 2	*L*/*R* = 1	*L*/*R* = 2	*L*/*R* = 1	*L*/*R* = 2	*L*/*R* = 1	*L*/*R* = 2
[0°/90°]	HSDT [58]	0.0804	0.1556	0.0938	0.1726	0.1085	0.1928	0.0444	0.0921
	FSDT [58]	0.0791	0.1552	0.0893	0.1697	0.1002	0.1876	0.0435	0.0914
	CST [58]	0.0866	0.1630	0.1152	0.1841	0.1048	0.2120	0.0480	0.0938
	FSDT [59]	0.0766	0.1519	0.0823	0.1661	0.0982	0.1737	0.0396	0.0872
	FSDT [57]	0.0881	0.1578	0.0921	0.1639	0.0982	0.1738	0.0396	0.0872
	WBM	0.0884	0.1581	0.0908	0.1631	0.0962	0.1723	0.0397	0.0873
[0°/90°/0°]	HSDT [58]	0.1007	0.1777	0.1087	0.1972	0.1192	0.2191	0.0506	0.0995
	FSDT [58]	0.1004	0.1779	0.1036	0.1945	0.1093	0.2129	0.0495	0.0988
	CST [58]	0.1479	0.2073	0.1850	0.2662	0.2049	0.3338	0.0669	0.1099
	FSDT [59]	0.0996	0.1722	0.1025	0.1950	0.1083	0.2083	0.0483	0.0914
	FSDT [57]	0.0996	0.1726	0.1028	0.1991	0.1086	0.2084	0.0483	0.0912
	WBM	0.0967	0.1706	0.0993	0.2043	0.1042	0.2017	0.0472	0.0907

**Table 3:** Frequency parameters $\Omega =\omega h\sqrt{\rho /G_{12}}/\pi$ for two types of cross-ply composite laminated cylindrical shells with different thickness to radius ratios under simply-supported boundary conditions (*R* = 1 m, *L/R* = 0.1, *E*_2_ = 1 GPa, *E*_1_/*E*_2_ = 40, *μ*_12_ = 0.25, *G*_12_ = 0.6*E*_2_, *G*_13_ = 0.5*E*_2_, *G*_13_ = 0.5*E*_2_, *ρ* = 1600 kg/m^3^).

*h*/*R*	[0°/90°/90°/0°]					[90°/0°/0°/90°]				
	WBM	Ref. [57]	Error	Ref. [59]	Error	WBM	Ref. [57]	Error	Ref. [59]	Error
0.1	0.0638	0.0639	−0.17%	0.0640	−0.32%	0.0531	0.0533	−0.38%	0.0531	0.00%
	0.0656	0.0657	−0.17%	0.0657	−0.17%	0.0591	0.0592	−0.24%	0.0591	−0.07%
	0.0789	0.0789	−0.05%	0.0789	−0.05%	0.0709	0.0710	−0.14%	0.0709	0.00%
0.2	0.1586	0.1588	−0.14%	0.1589	−0.20%	0.1332	0.1335	−0.22%	0.1333	−0.07%
	0.1676	0.1678	−0.15%	0.1683	−0.44%	0.1527	0.1528	−0.06%	0.1527	0.01%
	0.1726	0.1727	−0.07%	0.1726	−0.01%	0.1590	0.1593	−0.18%	0.1592	−0.12%
0.3	0.2539	0.2542	−0.11%	0.2546	−0.27%	0.2272	0.2275	−0.12%	0.2273	−0.03%
	0.2669	0.2670	−0.03%	0.2669	0.01%	0.2429	0.2430	−0.03%	0.2428	0.05%
	0.2785	0.2788	−0.11%	0.2797	0.43%	0.2697	0.2701	−0.14%	0.2699	−0.07%

**Table 4:** Frequency parameters $\Omega =\omega R\sqrt{\rho /E_{2}}$ for a three-layered cross-ply [0°/90°/0°] composite laminated cylindrical shell under simply-supported boundary conditions (*E*_2_ = 2 GPa, *E*_1_/*E*_2_ = 25, *μ*_12_ = 0.25, *G*_12_ = 0.5*E*_2_, *G*_13_ = 0.5*E*_2_, *G*_23_ = 0.2*E*_2_, *ρ* = 1600 kg/m^3^, *m* = 1).

*h*/*R*	*L*/*R* = 1		*L*/*R* = 2		*L*/*R* = 3		*L*/*R* = 4	
	*n* = 1	*n* = 2	*n* = 1	*n* = 2	*n* = 1	*n* = 2	*n* = 1	*n* = 2
0.05	1.54962	1.12747	0.78125	0.51865	0.52133	0.33977	0.39057	0.25367
0.06	1.58005	1.18476	0.78545	0.53046	0.52261	0.34539	0.39112	0.25800
0.07	1.61120	1.24206	0.79021	0.54365	0.52408	0.35183	0.39177	0.26299
0.08	1.64201	1.29756	0.79546	0.55794	0.52575	0.35899	0.39250	0.26857
0.09	1.67168	1.35011	0.80111	0.57306	0.52760	0.36680	0.39331	0.27468
0.1	1.69971	1.39908	0.80707	0.58877	0.52961	0.37516	0.39421	0.28127

**Table 5:** Frequency parameters $\Omega =\omega R\sqrt{\rho /E_{2}}$ for a three-layered cross-ply [0°/90°/0°] composite laminated cylindrical shell with clamped boundary conditions (*E*_2_ = 2 GPa, *E*_1_/*E*_2_ = 25, *μ*_12_ = 0.25, *G*_12_ = 0.5*E*_2_, *G*_13_ = 0.5*E*_2_, *G*_23_ = 0.2*E*_2_, *ρ* = 1600 kg/m^3^, *m* = 1).

*h*/*R*	*L*/*R* = 1		*L*/*R* = 2		*L*/*R* = 3		*L*/*R* = 4	
	*n* = 1	*n* = 2	*n* = 1	*n* = 2	*n* = 1	*n* = 2	*n* = 1	*n* = 2
0.05	1.74397	1.45928	0.82781	0.60980	0.54152	0.37875	0.40177	0.27465
0.06	1.79223	1.53994	0.84181	0.63996	0.54739	0.39300	0.40492	0.28362
0.07	1.83113	1.60423	0.85551	0.66928	0.55335	0.40776	0.40814	0.29316
0.08	1.86213	1.65516	0.86864	0.69708	0.55934	0.42277	0.41142	0.30314
0.09	1.88682	1.69562	0.88099	0.72294	0.56529	0.43778	0.41473	0.31345
0.1	1.90656	1.72802	0.89243	0.74672	0.57113	0.45260	0.41806	0.32398

**Table 6:** Frequency parameters $\Omega =\omega L^{2}\sqrt{\rho /E_{2}}/h$ for two types of cross-ply composite laminated cylindrical shells with classical boundary conditions (*L*/*R* = 4, *h*/*R* = 0.1, *E*_2_ = 2 GPa, *E*_1_/*E*_2_ = 25, *μ*_12_ = 0.25, *G*_12_ = 0.5*E*_2_, *G*_13_ = 0.5*E*_2_, *G*_23_ = 0.2*E*_2_, *ρ* = 1500 kg/m^3^, *m* = 1).

Layer-	*n*	SD-SD		S-S		C-C	
Type		Ref. [57]	WBM	Ref. [57]	WBM	Ref. [57]	WBM
[0°/90°/0°]	1	61.94	61.939	63.069	63.074	66.887	66.889
	2	42.76	42.739	44.99	45.003	51.846	51.837
	3	55.85	55.803	57.428	57.443	63.007	62.979
	4	92.309	92.249	93.101	93.108	96.611	96.569
[0°/90°]	1	59.523	59.523	62.065	62.069	62.677	62.676
	2	43.199	43.205	47.847	47.854	48.488	48.488
	3	73.147	73.145	75.891	75.888	76.138	76.13
	4	128.58	128.56	130.02	130.009	130.13	130.112

**Table 7:** Frequency parameters $\Omega =\omega L^{2}\sqrt{\rho /E_{2}}/h$ for two types of cross-ply composite laminated cylindrical shells with elastic boundary conditions (*L*/*R* = 4, *h*/*R* = 0.1, *E*_2_ = 2 GPa, *E*_1_/*E*_2_ = 25, *μ*_12_ = 0.25, *G*_12_ = 0.5*E*_2_, *G*_13_ = 0.5*E*_2_, *G*_23_ = 0.2*E*_2_, *ρ* = 1500 kg/m^3^, *m* = 1).

Layer-	*n*	EC1-EC1		EC2-EC2		EC3-EC3		EC4-EC4	
Type		Ref. [57]	WBM	Ref. [57]	WBM	Ref. [57]	WBM	Ref. [57]	WBM
[0°/90°/0°]	1	65.844	65.788	65.767	62.014	60.544	65.508	59.906	58.511
	2	50.056	50.019	51.223	51.279	50.187	49.273	49.672	49.629
	3	61.725	61.682	62.916	62.893	61.619	59.397	61.709	61.667
	4	95.949	95.902	96.592	96.551	95.548	102.61	95.949	95.902
[0°/90°]	1	61.265	61.167	56.838	55.912	62.231	62.15	55.912	54.997
	2	46.054	46.02	47.977	47.981	48.019	47.859	45.792	45.772
	3	74.745	74.738	76.084	76.083	75.941	75.817	74.743	74.737
	4	129.44	129.428	130.12	130.104	130.03	129.929	129.44	129.425

**Table 8:** The frequency parameters $\Omega =\omega L^{2}\sqrt{\rho /E_{2}}/h$ for a three-layered cross-ply [0°/90°/0°] composite laminated cylindrical shell with S-elastic boundary conditions, one displacement is under elastic restrained and others are free (*L*/*R* = 4, *h*/*R* = 0.1, *E*_2_ = 2 GPa, *E*_1_/*E*_2_ = 25, *μ*_12_ = 0.25, *G*_12_ = 0.5*E*_2_, *G*_13_ = 0.5*E*_2_, *G*_23_ = 0.2*E*_2_, *ρ* = 1500 kg/m^3^).

Spring Stiffness	*K_u_*			*K_v_*			*K_w_*			*K_ϕx_*			*K_ϕ__θ_*		
	*n* = 1	*n* = 2	*n* = 3	*n* = 1	*n* = 2	*n* = 3	*n* = 1	*n* = 2	*n* = 3	*n* = 1	*n* = 2	*n* = 3	*n* = 1	*n* = 2	*n* = 3
0	29.069	56.813	142.978	29.069	56.813	142.978	29.069	56.813	142.978	29.069	56.813	142.978	29.069	56.813	142.978
10^1^	29.069	56.813	142.978	29.069	56.813	142.978	29.069	56.813	142.978	29.069	56.813	142.978	29.069	56.813	142.978
10^2^	29.069	56.813	142.978	29.069	56.813	142.978	29.069	56.813	142.978	29.069	56.813	142.978	29.069	56.813	142.978
10^3^	29.069	56.813	142.978	29.069	56.813	142.978	29.069	56.813	142.978	29.069	56.813	142.978	29.069	56.813	142.978
10^4^	29.069	56.813	142.978	29.075	56.814	142.978	29.075	56.819	142.981	29.069	56.813	142.976	29.069	56.815	142.982
10^5^	29.069	56.813	142.978	29.131	56.829	142.981	29.131	56.876	143.005	29.069	56.811	142.958	29.069	56.837	143.024
10^6^	29.072	56.814	142.980	29.684	56.971	143.008	29.682	57.416	143.251	29.069	56.756	142.339	29.069	57.171	143.694
10^7^	29.097	56.825	142.996	34.324	58.233	143.288	34.122	60.798	145.643	29.069	56.854	143.276	29.069	55.922	141.734
10^8^	29.303	56.917	143.145	50.759	63.136	145.984	47.642	64.640	149.485	29.069	56.848	143.238	29.069	56.152	141.954
10^9^	29.975	57.257	143.872	59.203	65.439	149.834	53.982	65.281	150.110	29.069	56.847	143.235	29.069	56.168	141.973
10^10^	30.339	57.469	144.544	60.257	65.709	150.405	54.810	65.347	150.173	29.069	56.847	143.235	29.069	56.170	141.975
10^11^	30.392	57.502	144.669	60.364	65.737	150.462	54.895	65.353	150.179	29.069	56.847	143.235	29.069	56.170	141.975
10^12^	30.398	57.506	144.683	60.375	65.739	150.468	54.904	65.354	150.180	29.069	56.847	143.235	29.069	56.170	141.975

**Table 9:** The frequency parameters $\Omega =\omega L^{2}\sqrt{\rho /E_{2}}/h$ for a three-layered cross-ply [0°/90°/0°] composite laminated cylindrical shell with S-elastic boundary conditions, one displacement is under elastic restrained and others are free (*L*/*R* = 4, *h*/*R* = 0.1, *E*_2_ = 2 GPa, *E*_1_/*E*_2_ = 25, *μ*_12_ = 0.25, *G*_12_ = 0.5*E*_2_, *G*_13_ = 0.5*E*_2_, *G*_23_ = 0.2*E*_2_, *ρ* = 1500 kg/m^3^).

Spring Stiffness	*K_u_*			*K_v_*			*K_w_*			*K_ϕx_*			*K_ϕ__θ_*		
	*n* = 1	*n* = 2	*n* = 3	*n* = 1	*n* = 2	*n* = 3	*n* = 1	*n* = 2	*n* = 3	*n* = 1	*n* = 2	*n* = 3	*n* = 1	*n* = 2	*n* = 3
0	60.837	66.299	141.103	59.262	67.658	141.619	62.532	67.706	141.578	62.247	67.373	141.437	62.686	67.847	141.630
10^1^	60.837	66.299	141.103	59.262	67.658	141.619	62.532	67.706	141.578	62.247	67.373	141.437	62.686	67.847	141.630
10^2^	60.837	66.299	141.103	59.262	67.658	141.619	62.532	67.706	141.578	62.247	67.373	141.437	62.686	67.847	141.630
10^3^	60.837	66.299	141.103	59.262	67.658	141.619	62.532	67.706	141.578	62.247	67.373	141.437	62.686	67.847	141.630
10^4^	60.837	66.299	141.103	59.262	67.658	141.619	62.532	67.706	141.578	62.246	67.371	141.436	62.686	67.847	141.630
10^5^	60.837	66.300	141.103	59.263	67.658	141.619	62.532	67.707	141.578	62.231	67.352	141.426	62.686	67.847	141.630
10^6^	60.840	66.301	141.103	59.273	67.658	141.619	62.532	67.707	141.578	62.007	67.004	141.227	62.686	67.847	141.630
10^7^	60.871	66.318	141.107	59.368	67.660	141.619	62.537	67.712	141.580	62.858	67.987	141.676	62.686	67.848	141.630
10^8^	61.128	66.464	141.145	60.091	67.676	141.619	62.571	67.750	141.597	62.698	67.858	141.634	62.686	67.848	141.630
10^9^	62.042	67.140	141.349	61.872	67.757	141.623	62.651	67.822	141.623	62.687	67.848	141.631	62.686	67.848	141.630
10^10^	62.592	67.727	141.576	62.582	67.832	141.629	62.681	67.844	141.629	62.686	67.848	141.630	62.686	67.848	141.630
10^11^	62.676	67.835	141.624	62.675	67.846	141.630	62.685	67.847	141.630	62.685	67.847	141.630	62.686	67.848	141.630
10^12^	62.684	67.846	141.630	62.684	67.847	141.630	62.685	67.847	141.630	62.685	67.847	141.630	62.686	67.848	141.630
